# Single Walled Carbon Nanotubes Exhibit Dual-Phase Regulation to Exposed Arabidopsis Mesophyll Cells

**DOI:** 10.1007/s11671-010-9799-3

**Published:** 2010-09-28

**Authors:** Hengguang Yuan, Shanglian Hu, Peng Huang, Hua Song, Kan Wang, Jing Ruan, Rong He, Daxiang Cui

**Affiliations:** 1 College of Life Science and Engineering, Southwest University of Science and Technology, 621002 Mianyang, People's Republic of China; 2 Department of Bio-Nano Science and Engineering, National Key Laboratory of Nano/Micro Fabrication Technology, Key Laboratory for Thin Film and Microfabrication of Ministry of Education, Institute of Micro-Nano Science and Technology, Shanghai Jiao Tong University, 800 Dongchuan Road, 200240 Shanghai, People's Republic of China

**Keywords:** Single walled carbon nanotube, Arabidopsis mesophyll cells, Cellular uptake, Toxicity, Necrosis, Apoptosis, ROS

## Abstract

Herein we are the first to report that single-walled carbon nanotubes (SWCNTs) exhibit dual-phase regulation to Arabidopsis mesophyll cells exposed to different concentration of SWCNTs. The mesophyll protoplasts were prepared by enzyme digestion, and incubated with 15, 25, 50, 100 μg/ml SWCNTs for 48 h, and then were observed by optical microscopy and transmission electron microscopy, the reactive oxygen species (ROS) generation was measured. Partial protoplasts were stained with propidium iodide and 4'-6- diamidino-2-phenylindole, partial protoplasts were incubated with fluorescein isothiocyanate-labeled SWCNTs, and observed by fluorescence microscopy. Results showed that SWCNTs could traverse both the plant cell wall and cell membrane, with less than or equal to 50 μg/ml in the culture medium, SWCNTs stimulated plant cells to grow out trichome clusters on their surface, with more than 50 μg/ml SWCNTs in the culture medium, SWCNTs exhibited obvious toxic effects to the protoplasts such as increasing generation of ROS, inducing changes of protoplast morphology, changing green leaves into yellow, and inducing protoplast cells' necrosis and apoptosis. In conclusion, single walled carbon nanotubes can get through Arabidopsis mesophyll cell wall and membrane, and exhibit dose-dependent dual-phase regulation to Arabidopsis mesophyll protoplasts such as low dose stimulating cell growth, and high dose inducing cells' ROS generation, necrosis or apoptosis.

## Introduction

Carbon nanotubes, as a class of stiff, stable and hollow nanomaterials with unique physical and chemical properties, have been being actively explored potential in applications such as biomedical engineering, bioelectronics, etc. [1-5]. However, carbon nanotubes' biosafety cause more and more attention from government and people. Current researches mainly focus on two aspects, one is to study the detailed mechanisms of carbon nanotubes' cytotoxicity and their interaction with mammalian cells or plant cells in natural environment, the other is to study the methods of reducing carbon nanotubes' toxicity or enhancing their biocompatibility by surface functionalization [6-8]. Up to date, few data is closely associated with the effects of single walled carbon nanotubes (SWCNTs) on the plant cells in natural environment [9]. Therefore, to investigate the potential effects of SWCNTs on the plant cells in the natural environment is very necessary. In our previous studies, we observed that SWCNTs could inhibit the growth of human HEK293 cells and human fibroblast cells in dose- and time-dependent manner [10,11], and could induce the apoptosis of embryonic stem cells [12]; antisense oligonucleotides conjugated SWCNTs could enter into HL-60 cytoplasm and kill tumor cells [13]; dendrimer-modified multiwall carbon nanotubes could efficiently deliver genes or drugs into tumor cells [14]. In addition, SWCNTs have been reported to exhibit strong antimicrobial activity [15]; biomolecules-conjugated SWCNTs could get through BY-2 cell walls, and exhibited little toxicity to BY-2 cells [16], carbon nanotubes could penetrate plant seed coat and dramatically affect seed germination and plant growth [17]. So far the plant cells exposed to SWCNTs appear what changes, such as cellular uptake, mitochondrial function, cellular LDH release, apoptosis signaling pathway, reactive oxygen species (ROS) generation and pro-inflammatory cytokine release, etc., are not clarified well.

Arabidopsis, as an important model in plant biology and genetics, has been broadly investigated [18-20]. Many findings with direct relation to human health and diseases have been elaborated by using Arabidopsis thaliana, and several processes important to human biology are more easily studied in this versatile model plant [21]. Therefore, investigating the influence of SWCNTs on Arabidopsis thaliana owns broad representative.

In this study, we selected Arabidopsis thaliana as research target, and investigated the effects of highly purified SWCNTs on Arabidopsis mesophyll cells with the aim of observing active reaction and possible mechanism of plant cells exposed to different dose of SWCNTs.

## Experiments

### Source of Materials

SWCNTs were purchased from Carbon Nanotechnologie, Inc. (CAS no.308068-56-6), their purity was 99.8%. Arabidopsis thaliana was provided by prof. Dabing Zhang in School of Life Science and Biotechnology of Shanghai Jiao Tong University. Mesophyll cells were freshly isolated from the leaves of wild-type Col-0 (top) growing on the soil. Other reagents such as fluorescein isothiocyanate (FITC) were purchased from Sigma–Aldrich, USA.

### Characterization of SWCNTs

SWCNTs were characterized by Atom Force Microscopy [13], dynamic light scattering for zeta potential [22], and Raman spectroscopy. Raman spectra were recorded on a Jasco Raman spectrometer. The instrument is equipped with Olympus confocal microscope. Excitation wavelengths of 488, 532, and 785 nm were used.

### Protoplasts Preparation and Culture

The protoplasts were prepared by enzyme digestion method [23,24]. The concrete steps are as follows: firstly, obtain 20 leaves; then, cut 0.5–1 mm leaf strips from middle part of a leaf using a fresh sharp razor blade without tissue crushing at the cutting site; put 20 leaves into 10 ml enzyme solution, which was prepared as follows: the 1 ml 0.2 M 4-morpholineethanesulfonic acid (MES) was added into 0.15 g cellulase R10, 0.04 g macerozyme R10, 5 ml 0.8 M mannitol and 100 μl 2 M KCl; then the solution was heated up to 55°C for 10 min, and then was cooled down to 25°C, finally, was added into 100 μl 1 M CaCl_2_, 25 μl β-mercaptoethanol and 100 μl 10% bovine serum albumin (BSA), resultant final enzyme solution was filtered through a 0.45 μm syringe filter device into the Petri dish. Vacuum infiltrated leaf strips for 30 min in the dark using a desiccator, then continued the digestion for 3 h at room temperature under the condition of dark and no-shaking. Then checked the release of protoplasts in the solution under the microscope (Nikon TS100), and diluted the enzyme/protoplast solution with an equal volume of W5 solution composed of 2 mM MES (pH 5.7), 154 mM NaCl, 125 mM CaCl_2_ and 5 mM KCl. Then, washed a 75 μm nylon mesh with W5 solution, and filtered the enzyme solution including the protoplasts, and then centrifuged the filtered products at 600 rpm for 2 min, then removed the supernatant as much as possible, the final yield was the protoplasts. Then, the 6-well microplate was firstly washed by 5%(vol/vol) sterile BSA, and was added into 2 aliquots (0.5 ml each) of prepared Arabidopsis mesophyll protoplasts, then, the protoplasts was gently suspended with 1 ml washing and incubation (WI) solution (4 mM MES pH 5.7, 0.5 M mannitol and 20 mM KCl), and was incubated at 22°C.

### Protoplasts Incubated with SWCNTs

The prepared protoplasts were cultured in 6-well microplate with WI solution. Then 15, 25, 50, 100 μg/ml SWCNTs solution were added into one well, and a control well without SWCNTs was set up. Meanwhile, the protoplasts were observed by microscope (Nikon TS100), one time per 8 h, until 48 h. FITC-labeled SWCNTs was prepared according to our previous reports [13], and incubated with the prepared protoplasts in the culture microplate.

### Observation of Mesophyll Cells and Protoplasts

Arabidopsis was cultured for 24 days, then the sterilized tweezers were used to slit wild-type lower epidermis of Arabidopsis leaves, then 50 μg/ml SWCNTs were dropped on the surface of mesophyll cells, kept for 2 h, and then slowly washed with sterilized water for three times, partial collected cells were embedded and made into transmission electron microscope (TEM) specimens, and then observed via a JEM-2100 electron microscope.

### The Apoptosis Analysis of the Protoplasts Incubated with SWCNTs

The protoplasts were incubated with 50 μg/ml SWCNTs for 40 h, then, the protoplasts were stained with 50 μg/ml propidium iodide (PI) for 15 min and counter-stained with 3 μg/ml DAPI(4'-6-diamidino-2-phenylindole) for 5 min in the dark, finally those samples were observed by fluorescence microscope (Olympus IX71), and analyzed by flow cytometer (FCM).

### Determination of ROS

The ROS assay was finished according to the manufacturer's manual. Briefly, the prepared protoplasts were incubated at 37°C for 24 h. Then, the culture medium was aspirated and the protoplasts were washed with Dulbecco PBS(DPBS) followed by the addition of 1 ml of fresh culture medium containing 10 μM carboxy-DCFDA (C-400, molecular probes, CA, USA) dissolved in DMSO. After the protoplasts were incubated for 15 min in a CO_2_ incubator, 10 μl of test solution was added to the protoplasts. Hydrogen peroxide (H_2_O_2_) was used as a positive control stimulus. Following exposure for 60 min, the protoplasts were washed by DPBS once and harvested with trypsin–EDTA. Finally, the protoplasts were suspended with 0.3 ml of 10% FBS in DPBS and passed through nylon mesh. The protoplasts were subjected to flow cytometry (FACSCaliburTM, BD company, USA) until 20,000 events were recorded.

### Data Analysis

All data are presented in this paper as means result ± SD. Statistical differences were evaluated using the *t*-test and considered significance at *P* < 0.05 level. All figures shown in this article were obtained from three independent experiments with similar results.

## Results

### Characterization of SWCNTs

**Figure 1:**
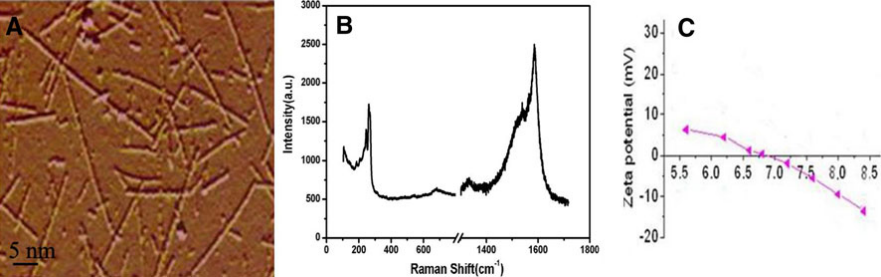
**a AFM image of SWCNTs**; **b** Rama spectra of SWCNTs; **c** Zeta Potential of SWCNTs.

As shown in Figure 1a, prepared SWCNTs were very pure, and well dispersed, Figure 1b was the Raman spectra of SWCNTs, showing that the prepared SWCNTs were of high purity. Figure 1c showed the Zeta potential of SWCNTs in water solution.

### Observation of SWCNTs Penetrating Mesophyll Cell Walls

**Figure 2:**
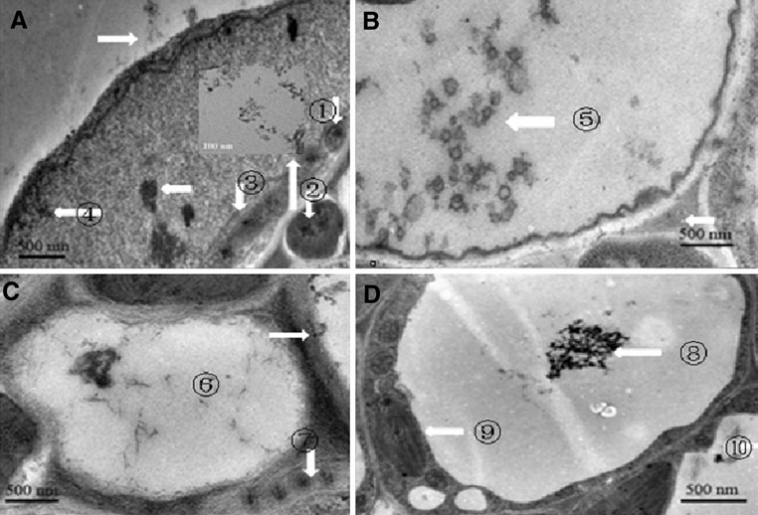
**TEM images of SWCNTs entered into *Arabidopsis* mesophyll cells**. **a** Some of the SWCNTs attached to the surface of cell wall, some crossed the membrane and located in the inner of cell membrane, mitochondria ①, nucleus ②, the inset is magnified image of SWCNTs inside the nucleus; B: SWCNTs located inside the vacuole; C: SWCNTs located in the vacuole ⑥ and chloroplast ⑦; D: aggregated SWCNTs located in the vacuole, ⑧⑨ ⑩ showed SWCNTs in different position.

**Figure 3:**
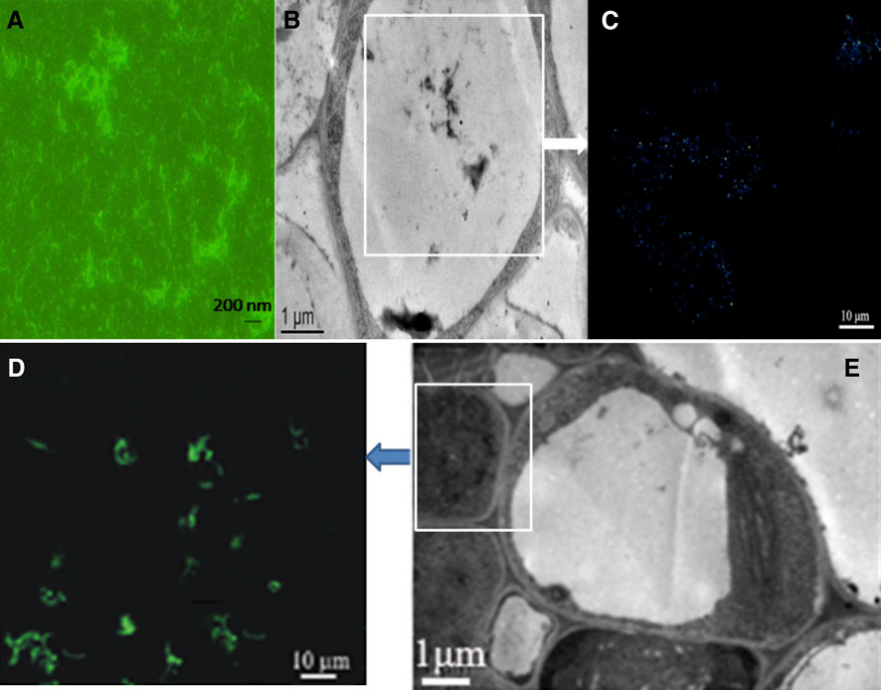
**FCNTs located inside plant cells**. **a** Prepared FCNT; **b** TEM image of FCNTs located inside the vacuole; **c** Fluorescent microscope image of FCNTS inside the vacuole; **e** TEM picture of FCNTs inside nucleus, **d** fluorescent microscope image of FCNTs inside nucleus.

As shown in Figure 2a–d, a few SWCNTs attached to the surface of plant cell wall, a lot of SWCNTs penetrated cell wall to enter inside the plant cell. Figure 2a ① showed the SWCNTs located in mitochondria, Figure 2a ② showed the SWCNTs located in nucleus, the inset was the magnified picture that clearly showed the existences of SCWNTs, Figure 2a ③, c ⑦ and d ⑨ showed the SWCNTs inside the chloroplast, Figure 2a ④ showed the SWCNTs just got through cell wall, Figure 2b ⑤, and c ⑥ and d ⑧ showed the SWCNTs inside the vacuole. In order to confirm that SWCNTs could penetrate cell wall to enter into the inner of plant cell, we used FITC-labeled SWCNTs (FCNTs) to incubate with plant cells. Figure 3a showed the prepared FCNTs, Figure 3b, c showed the FCNTs inside the vacuole, Figure 3d, e clearly showed the FCNTs inside the nucleus. All these data mentioned above fully demonstrate that SWCNTs can enter into *Arabidopsis* mesophyll cells. It is the first to report that SWCNTs was confirmed to get through the *Arabidopsis* mesophyll cell wall, and enter into the inner organelles of the mesophyll cell. We also put the *Arabidopsis* mesophyll cells into different temperature solution such as 4, 25 and 40°C, and observed the fluorescent signal intensity, we did not observe the obvious changes of fluorescent intensity (data not shown), which highly suggest that SWCNTs likely get through plant cell wall and membrane by non-energy dependent endocytosis manner.

### Effects of SWCNTs on Morphology of *Arabidopsis* Mesophyll Protoplasts

**Figure 4:**
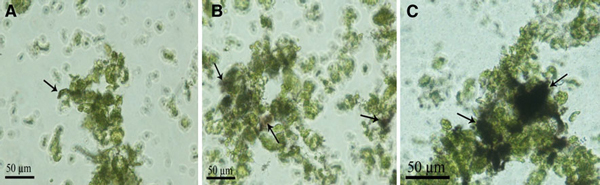
**Optical microscope images of protoplasts incubated with 50 μg/ml SWCNTs**. **a** Protoplasts dispersed well; **b** partial protoplasts aggregated together; **c** protoplasts aggregated together.

As shown in Figure 4, under the condition of 50 or 100 μg/ml SWCNTs in culture media, SWCNTs actively attached to the surface of the protoplasts, some of the SWCNTs aggregated together, all the protoplasts became small in size and appeared wrinkly as the culture time increased, which highly suggested that SWCNTs can change the morphology of protoplasts.

**Figure 5:**
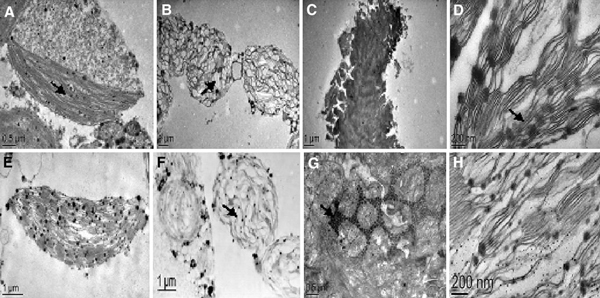
**TEM images of protoplasts**. **a** The chloroplasts in normal protoplasts; **e** the chloroplasts of protoplasts treated with SWCNTs; **b** fluffy chloroplasts in control protoplasts; **f** fluffy chloroplasts in the protoplasts treated with SWCNTs; **c** the part structure of normal protoplasts; **g** the part structure of protoplasts treated with SWCNTs; **d** the filament structure in the chloroplasts; **h** the loosen filament structure in the chloroplasts in protoplasts treated with SWCNTs, all *arrows* indicated the location of SWCNTs.

We also observed the morphological changes of chloroplasts in protoplasts incubated with SWCNTs for 48 h. As shown in Figure 5e–h, SWCNTs could enter into chloroplasts, and caused the chloroplasts looseness of structure, which was very obvious in Figure 5h.

**Figure 6:**
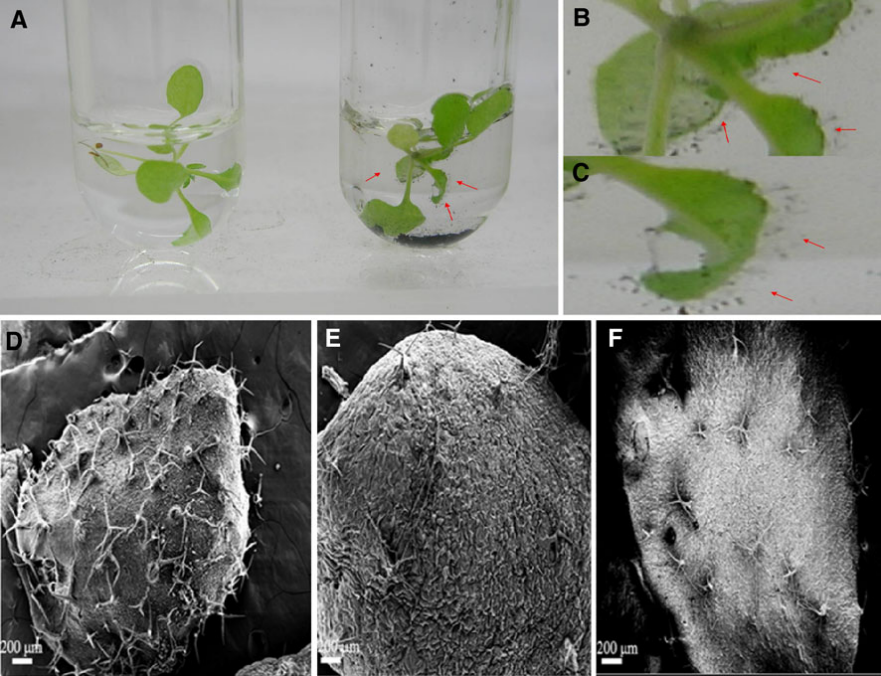
**Optical images and SEM images of Arabidopsis mesophyll cells exposed to SWCNTs**. **a** Plant cells treated without or with 15 μg/ml SWCNTs; **b** and **c** plant cells treated with 15, 25 μg/ml of SWCNTs for 24 h. **d** Plant cells treated with 25 μg/ml of SWCNTs for 48 h; **e** plant cells without exposed SWCNTs as control; **f** plant cells treated with 15 μg/ml of SWCNTs for 24 h.

However, surprisingly, we observed that, as shown in Figure 6, under the condition of 25 μg/ml SWCNTs and 15 μg/ml SWCNTs in culture media, there existed many new-born trichome clusters on the surface of the plant leaves, the trichome clusters on the surface of leaves treated with 25 μg/ml SWCNTs was markedly more than those treated with 15 μg/ml SWCNTs, compared with control, there existed statistical difference among their amounts of three groups (*P* < 0.05). Therefore, we consider that low dose of SWCNTs can speed up the trichome development.

### Effects of SWCNTs on the Viability of Protoplasts

**Figure 7:**
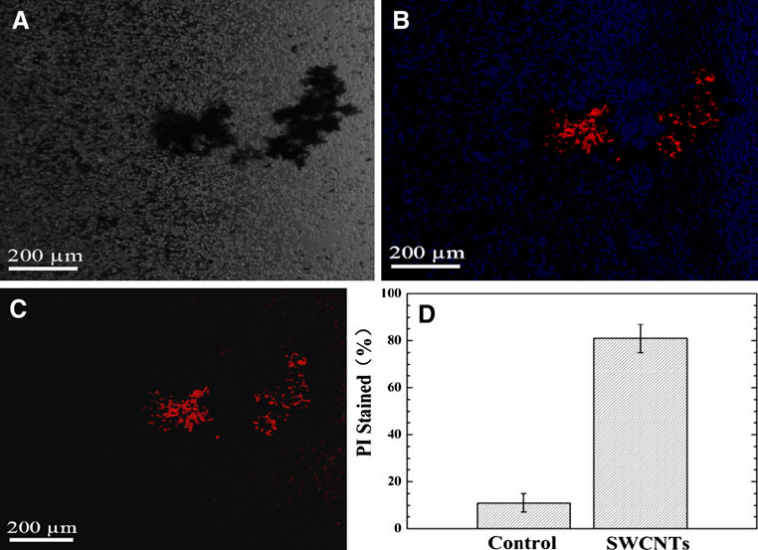
**Protoplast viability analysis after exposure to 50 μg/ml SWCNTs in a WI solution**. **a** Microscopic image of SWCNTs aggregates. **b** Fluorescence microscope image of total protoplasts (protoplasts stained with PI and DAPI). **c** Fluorescence microscope image of dead protoplasts (stained with PI only). **d** Fluorescence-based assay showed that the inhibition activity of SWCNTs on the protoplasts as the percent of protoplasts stained with PI or the percent of loss of viability.

The effects of SWCNTs on the viability of protoplasts incubated with 50 μg/ml SWCNTs were investigated. When the protoplasts were treated with 50 μg/ml SWCNTs for 40 h, we used propidium iodide (PI) and 4'-6-diamidino-2-phenylindole (DAPI) to stain the protoplasts, as shown in Figure 7b, c, we observed that SWCNTs aggregated together, protoplasts also wrapped around the aggregated SWCNTs, and a lot of protoplasts were dead (red color). As shown in Figure 7d, after the protoplasts were treated with SWCNTs for 48 h, the death cells reached 80% or so, appeared yellow leaves, compared with the control group, there exists statistical difference between two groups, *P* < 0.05. In control group, protoplasts often were live in WI solution for 4 days, our results showed that 50 μg/ml SWCNTs in a WI solution could speed up the death of protoplasts in WI solution, inducing green leaves into yellow leaves similar phenomena was also observed for those protoplasts treated with 100 μg/ml SWCNTs in a WI solution.

We also used flow cytometry (FCM) to analyze these cells treated with 50 μg/ml SWCNTs, results showed that 70% or so cells exhibited apoptosis properties (data not shown). Therefore, more than 50 μg/ml SWCNTs could inhibit the growth of protoplasts by inducing apoptosis means, similar to our previous reports [10,11].

However, after the protoplasts treated with 25 or 15 μg/ml SWCNTs for 24 h, these protoplasts grew very well, and which fully showed that low dose of SWCNTs can improve the survivorship and development of protoplasts.

### Formation of ROS

**Figure 8:**
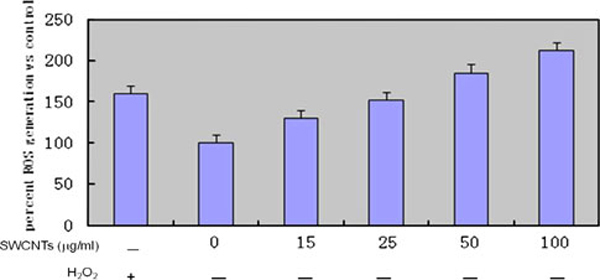
**Intracellular production of ROS in *Arabidopsis* mesophyll cells**. Plant cells were incubated for 24 h without SWCNTs (negative control) or with 0, 15, 25, 50, 100 μg/ml of SWCNTs and stained with C-400. Fluorescent intensity was monitored by flow cytometry. The intracellular generation of ROS is expressed as the percentage of control. Hydrogen peroxide (H2O2) was used as a positive control. *Bars* indicate the mean ± SE of two independent experiments.

We investigated if SWCNTs causes ROS formation in the *Arabidopsis* mesophyll cells by using DCF(2',7'-dichlorofluorescin diacetate) fluorescence as a reporter of intracellular oxidant production. As shown in Figure 8, as the concentration of SWCNTs in medium increased, the gradually enhanced DCF response was observed after a 24-h exposure to SWCNTs at concentrations of 0, 15, 25, 50, 100 μg/ml, which highly suggest that highly purified SWCNTs can cause oxidative stress reaction of plant cells.

## Discussion

Up to date, the influence of nanomaterials on natural environment has become a hotspot. Carbon nanotubes, as one kind of promising nanomaterials, has been actively investigated potential application in biomedical engineering. However, so far the detailed mechanism of interaction between carbon nanotubes and the plant cells is still not well clarified.

In this study, we found that SWCNTs can get through the *Arabidopsis* mesophyll cell walls, and enter into intact *Arabidopsis* mesophyll cells, and then enter into the organelles such as chloroplast, vacuole, mitochondria, and nucleus, thus, SWCNTs may be a good molecular transporter in the plant world. At low dose of SWCNTs(less than 50 μg/ml) in culture media, SWCNTs can improve survivorship and development of plant cells, at high dose of SWCNTs in culture media (more than 50 μg/ml), SWCNTs exhibit obvious toxicity to *Arabidopsis* mesophyll protoplasts. The protoplasts gradually aggregated around SWCNTs, more and more wrinkles happened, as the incubation time of SWCNTs in WI solution with *Arabidopsis* mesophyll protoplasts increases, finally exhibited necrosis or apoptosis at high dose of SWCNTs. Our result also showed that SWCNTs can cause generation of a lot of intracellular reactive oxygen species (ROS) in plant cells incubated with different concentration of SWCNTs, which highly suggest that oxidative stress may be one of mechanism of active reaction of plant cells exposed to SWCNTs. Therefore, our results fully demonstrate that SWCNTs exhibit dual-phase regulation to plant cells in natural environment.

With the regard to the uptake mechanism of SWCNTs by plant cells, so far it is not clarified well. It is well known that uptake of carbon nanotubes into mammalian cells is often by the endocytosis and insertion/diffusion [24]. It has been reported two possible pathways of SWCNTs to enter into cells are as follows: energy-dependent phagocytosis or endocytosis and passive diffusion across lipid bilayers [28]. For plant cells, endocytosis pathway of extracellular molecules has also been investigated [30,31], for example, Liu et al. [16] reported that carbon nanotubes could be used as molecular transporters for walled plant cells and proposed that SWCNTs/fluorescein isothiocyanate was taken up by fluidic-phase endocytosis. In this study, we observed that SWCNTs primarily located within lysosomes, then entered into the cytoplasm and the nucleus, and aggregated together, we also observed that temperature almost did not affect the uptake of SWCNTs by plant cells, therefore, we consider that, for the plant cells like *Arabidopsis* mesophyll cells, SWCNTs enter into plant cells via non-energy dependent endocytosis pathway, which may be one kind of nanoscale capillary function, the van der Waals and hydrophobic forces are likely to be very important for the cellular uptake process of SWCNTs, similar to our previous report that DNA molecules entering into carbon nanotubes [29], further confirmation work is under way.

Regarding the toxicological mechanisms of carbon nanotubes, current reports mainly include three aspects such as oxidative stress, metal toxicity and physical piercing [25-27]. For microorganism, previous report showed that the *E. coli* underwent severe membrane disruption and subsequent loss of viability due to SWCNTs [15], metal toxicity was also an important toxicity mechanism, but highly purified SWCNTs were no residual metal catalysts in their tubes, which may be not the main toxicity mechanism. The induction of oxidative stress is the one of main toxicity mechanism of carbon nanotubes for mammalian cells [22-25]. For example, Tian et al. [11] reported SWCNTs also could damage the cytoarchitecture, fused with the plasma membrane, and caused cell damage through lipid peroxidation and oxidative stress. In our study, we also observed that highly purified SWCNTs can cause the production of ROS in plant cells, which shows that ROS may be main toxic mechanism of SWCNTs to exposed plant cells. Some studies show that the physical interaction between carbon nanotubes and cells, rather than oxidative stress, is the primary killing mechanism for mammalian cells [29,30]. For plant cells, physical interaction may be one of the main toxic mechanisms, which needs still further investigation.

In our study, in order to distinguish apoptosis of plant cells from death of plant cells, we used FCM to analyze the plant cell cycles, results showed that apoptosis cells reach 70% of total cells after incubation of plant cells with 50 μg/ml SWCNTs for 48 h. Programmed cell death (PCD) is an integral part of plant development and defense. Essential for normal functioning of plants, PCD may be found throughout the life cycle, from the fertilization of the ovule, to the formation of the xylem, to the death of the whole plant. Thus far, the fate of chloroplasts has been studied mainly during the developmental PCD of leaves that yellow at the end of the growing season. It is now widely accepted that the moment the cell dies during PCD is the time of vacuolar collapse, i.e., permabilization or rupture of the tonoplast. Organelles were degraded only after the rupture of the tonoplast, which releases hydrolases from the vacuole. The ER and Golgi bodies disappear, followed by the chloroplasts and mitochondria. The chloroplast matrix is degraded first, then the internal membrane structure [32]. Therefore, we think that PCD may be main toxic mechanisms of SWCNTs to plant cells. According to data above-mentioned, we suggest the possible interaction model between SWCNTs and *Arabidopsis* mesophyll cells as follows: the SWCNTs in water solution can attach to the surface of plant cells, and penetrate cell walls into plant organelles by non-energy dependent endocytosis pathway, mainly locate in the vacuole, chloroplast, mitochondria, and nucleus, SWCNTs in the mitochondria may disturb the energy-metabolism course of plant cells, SWCNTs in chloroplast may disturb the photosynthesis course, SWCNTs in nucleus may damage the function of nucleus, at the same time, plant cells appear active reactions such as the shrink of plant cells, with the aim of fighting against the foreign invaders, when the balance between survivals and death was damaged, finally result in the apoptosis of plant cells. However, as we observed, low dose of SWCNTs improve the development of trichome clusters [33,34] and growth of mesophyll cells [35,36], which highly suggest that plant cells may own powerful, unique protective function to fight against the foreign invaders. Although SWCNTs enter into important organs such as nucleus, chloroplast, mitochondria, etc., low dose of SWCNTs, as foreign invaders, can not completely damage the function of important organs due to powerful self-protection and repairing function of important organs [37], until reach the maximal dose inside local organs, exceed the permission limitation of plant cells, and finally result in marked toxicity to cells. Further work will focus on the molecular mechanism of active reaction of plant cells to SWCNTs.

## Conclusions

In conclusion, our study provides the direct proofs that SWCNTs traverse across both the plant cell wall and cell membrane, high dose of SWCNTs exhibit toxic effects on the protoplasts, inducing changes of protoplasts morphology and protoplasts apoptosis, causing the production of intracellular ROS in plant cells, low dose of SWCNTs improve the survivorship and development of plant cells. Although present mechanism is not clarified well, this phenomenon should be the first report, which owns great potential in applications such as use of low dose of SWCNTs as the accelerated reagents for plant cells' growth and development, use of high dose of SWCNTs as toxic reagents to damage bad plant cells. Our studies lay foundation for further investigation mechanism of interaction between SWCNTs and plant cells in nature environment.
